# Transcranial Random Noise Stimulation (tRNS) Shapes the Processing of Rapidly Changing Auditory Information

**DOI:** 10.3389/fncel.2017.00162

**Published:** 2017-06-08

**Authors:** Katharina S. Rufener, Philipp Ruhnau, Hans-Jochen Heinze, Tino Zaehle

**Affiliations:** Department of Neurology, Otto-von-Guericke UniversityMagdeburg, Germany

**Keywords:** transcranial random noise stimulation (tRNS), auditory processing, auditory temporal resolution, stochastic resonance, resonance frequency

## Abstract

Neural oscillations in the gamma range are the dominant rhythmic activation pattern in the human auditory cortex. These gamma oscillations are functionally relevant for the processing of rapidly changing acoustic information in both speech and non-speech sounds. Accordingly, there is a tight link between the temporal resolution ability of the auditory system and inherent neural gamma oscillations. Transcranial random noise stimulation (tRNS) has been demonstrated to specifically increase gamma oscillation in the human auditory cortex. However, neither the physiological mechanisms of tRNS nor the behavioral consequences of this intervention are completely understood. In the present study we stimulated the human auditory cortex bilaterally with tRNS while EEG was continuously measured. Modulations in the participants’ temporal and spectral resolution ability were investigated by means of a gap detection task and a pitch discrimination task. Compared to sham, auditory tRNS increased the detection rate for near-threshold stimuli in the temporal domain only, while no such effect was present for the discrimination of spectral features. Behavioral findings were paralleled by reduced peak latencies of the P50 and N1 component of the auditory event-related potentials (ERP) indicating an impact on early sensory processing. The facilitating effect of tRNS was limited to the processing of near-threshold stimuli while stimuli clearly below and above the individual perception threshold were not affected by tRNS. This non-linear relationship between the signal-to-noise level of the presented stimuli and the effect of stimulation further qualifies stochastic resonance (SR) as the underlying mechanism of tRNS on auditory processing. Our results demonstrate a tRNS related improvement in acoustic perception of time critical auditory information and, thus, provide further indices that auditory tRNS can amplify the resonance frequency of the auditory system.

## Introduction

Ongoing oscillatory activity in the gamma range is strongly associated with the processing of acoustic input at the level of the auditory cortex. In particular, auditory gamma activity seems to be especially relevant for the parsing and decoding of acoustic information taking place in a very short time range (Rosen, 1992).

This functional link between gamma oscillations in the auditory cortex and temporal acoustic processing is proposed to reflect the underlying mechanism of speech perception by analyzing fine grained information at the phonemic scale, such as formant transition or voicing (Poeppel, 2003; Morillon et al., 2010, 2012). In the speech signal, voicing is determined by the time-critical information of the Voice-onset-Time (VOT). The VOT—determining whether phonemes are perceived as voiced (/da/) or voiceless (/ta/)—has a critical time range of about 20–60 ms depending on the individual language (Lisker and Abramson, 1964). An appropriate temporal resolution of the auditory system is thus a crucial prerequisite to the successful encoding of the acoustic speech signal. The auditory cortex parses the incoming acoustic signal at its inherent frequency, i.e., the resonance frequency around 40 Hz (Zaehle et al., 2010a). Accordingly, the resonance frequency corresponds to linguistically relevant time units in the acoustic speech signal. Maladaptive auditory gamma activity should therefore affect the perception of short or fast changing acoustic information. In fact, patients suffering from dyslexia, a neuropsychological disorder typically characterized by decreased temporal resolution as evident in impaired VOT-discrimination ability (Breier et al., 2001), also show alterations in neural gamma oscillations (Lehongre et al., 2013; Goswami et al., 2014). Moreover, also in normal aging detrimental temporal resolution abilities in the auditory system (Walton, 2010) can be attributed to reduced auditory gamma activity (Jacobson et al., 2013; Rufener et al., 2015; Miraglia et al., 2016).

In this vein, Baltus and Herrmann (2015) recently demonstrated a functional link between auditory temporal resolution abilities and the individual “preferred” frequency of the auditory cortex. The close association between auditory perception and endogenous gamma frequency indicates that the frequency of an individuals’ oscillatory activation pattern i.e., the resonance frequency, determines the pace at which the auditory system optimally processes incoming sensory information. Thus, endogenous activity in the gamma range in auditory cortex regions is considered elementary for processing the acoustic properties in speech and non-speech signals.

With regards to the speech signal, in two former studies we provided systematic evidence for the functional relevance of auditory gamma activity in phoneme processing (Rufener et al., 2016a,b). By means of transcranial alternating current stimulation (tACS), we applied weak sinusoidal electrical currents at 40 Hz over bilateral auditory cortex regions in order to modulate endogenous gamma activity. We demonstrated that tACS in the gamma range shaped the individual phoneme categorization, demonstrating that rapid temporal information processing can be modulated via (frequency specific) 40 Hz-tACS. However, tACS-induced neuronal entrainment is supposed to be strongest when the external stimulation frequency is at or close to the network’s preferred frequency (Ali et al., 2013; Fröhlich, 2015), i.e., the systems resonance frequency but these resonance frequencies are highly variable across individuals (Zaehle et al., 2010a). Thus, tACS might be better suited to modulate those processes that are closely related to specific—*a priori* known or measurable—peak frequencies. While this holds true for alpha activity with typically clearly extractable peaks in the individual frequency spectra (Zaehle et al., 2010b; Neuling et al., 2013), the assessment of individual endogenous gamma band activity is not that straightforward. This might, at least in part, explain the broad range of diverging results from studies investigating the effect of tACS on (auditory) perception and the often rather limited statistical power of the reported effects (for a review see Zoefel and Davis, 2016).

A possible method to affect the resonance frequency of the auditory cortex is transcranial random noise stimulation (tRNS), which has been demonstrated to directly amplify individual gamma activity in the auditory cortex (Van Doren et al., 2014). In contrast to tACS, tRNS applies alternating electrical currents of different frequencies and amplitudes i.e., electrical white noise (for an overview, see Antal and Herrmann, 2016; Heimrath et al., 2016). Thus, tRNS allows for enhancing auditory gamma activity without prior knowledge of the individual endogenous gamma band frequency. Although the exact influence of tRNS on neurophysiology is not fully clear, stochastic resonance (SR) has been hypothesized as the underlying mechanisms of action. SR describes the phenomenon that the perception of a near threshold stimulus, i.e., a signal with a critical signal-to-noise-ratio (SNR) is enhanced if noise is presented in addition. For this positive effect noise needs to be presented at an optimal level. Crucially, no such effect is observable for stimuli with too low or too high SNR. Accordingly, the critical SNR between the acoustic information of the stimulus and the noise level of the system predicts detection rate. A tRNS induced improvement of the central nervous systems SNR and the sensitization of sensory processing might thus lead to enhanced perception (Moss et al., 2004).

In sum, there is ample evidence that gamma band activity at around 40 Hz is the inherent and dominant oscillatory pattern in the auditory cortex. These 40 Hz oscillations are functionally relevant for the processing of temporal features in the acoustic signal. While both tACS and tRNS can modulate auditory gamma activity, the latter allows for a frequency unspecific application without prior knowledge on the specific frequency of the target resonator. However, until today, no systematic investigation on the behavioral consequences of auditory tRNS has been conducted. In the present study we systematically investigated the consequences of modulating the auditory cortex by means of tRNS on behavioral as well as on electrophysiological measures. To assess individual acoustic processing, we utilized separate measures of temporal and spectral resolution. We hypothesized that auditory tRNS solely modulates temporal acuity via the synchronization of functionally relevant neural assemblies in the gamma range. Furthermore, since tRNS modulates the noise level in the auditory system, we assume to find a positive effect on the perception of near threshold stimuli only, while tRNS should not affect the detection of stimuli clearly below or above the perception threshold, representing low and high SNR, respectively. Finally, behavioral effects should be mirrored in stimulus-evoked brain response patterns.

## Materials and Methods

### Participants

Twenty healthy participants (10 female) in the age range of 20–35 years recruited via advertisement at the University of Magdeburg took part in this study. All participants were right handed as tested by the Edinburgh Handedness inventory (Oldfield, 1971), reported normal hearing acuity and had no history of neurologic or psychiatric disorders. All subjects gave written informed consent in accordance with the Declaration of Helsinki. The protocol was approved by the ethics committee of the University of Magdeburg.

### Assessment of the Detection Thresholds

Prior to each session, the participants underwent a threshold assessment of the individual gap detection (temporal resolution) and pitch discrimination (spectral resolution) abilities. Participants were instructed to listen carefully to all presented sounds and to solve the task as accurately as possible without any time constraints. No explicit feedback on the task performance was given. All participants performed the two threshold assessments (i.e., gap detection and pitch discrimination) repeatedly for 15 min. In order to avoid any order effects, the sequence was balanced between the participants, so that 50% started with the gap detection threshold (GDT) and the other half of the study sample started with the pitch discrimination threshold (PDT).

#### Gap Detection Threshold (GDT)

To study individual temporal processing abilities, we utilized a between-channel gap detection task (Zaehle et al., 2004). Performing such a task requires the perception of a short temporal gap between the offset of the leading element (i.e., a wideband noise burst with a length of 7 ms) and the onset of the trailing element (a band-passed noise centered on 1000 Hz and a width of 500 Hz with a length of 300 ms; Phillips et al., 1997, 1998). We assessed the individual GDT as a measure of auditory temporal resolution ability. The listener was presented with two streams of sounds, one of which comprised of a brief silent period (“gap”). The listener’s task was to identify the gap stimulus and to respond via mouse click. There were no time restrictions and the subject could repeatedly listen to the stimulus pairs until they felt certain in their decision. The first detectable stimulus was presented with an initial gap of 100 ms, which was then adjusted stepwise by an up/down staircase (Kesten, 1958): if the gap was identified correctly, the gap in the next trial was decreased (first step width: 12.5 ms, subsequently, the step width was divided by two until a minimum step width of 3.2 ms was reached), if the gap was identified incorrectly, the gap in the next trial was increased by the factor two (irrespective whether the last trial was solved correctly or incorrectly). The assessment was terminated after three reversals and a GDT was computed by the arithmetic mean of the last three reversals.

#### Pitch Discrimination Threshold (PDT)

The individual PDT was assessed using an analogous adaptive up/down staircase procedure. In each trial, the listener was presented with two sine wave tones, one of which was a standard tone of 1000 Hz and a second tone consisting of a different pitch (higher or lower). The two tones were presented in random order. The listener’s task was to identify the higher pitch tone (target tone) and the smallest detectable pitch difference was determined. There were no time restrictions the participants could repeatedly listen to the stimulus pairs until they felt safe in their decision. The first detectable stimulus was presented with the initial pitch difference of 100 Hz, which was then adjusted stepwise by an up/down staircase: if the target tone was identified correctly, the pitch difference in the next trail was decreased (first step width: 7.5 Hz, subsequently the step width was divided in half with every next step until a minimum step width of 1.9 Hz), if the target tone was identified incorrectly, the pitch difference was increased by the factor two of the last trial (irrespective whether this was solved correctly or incorrectly). The trials were terminated following three reversals and the PDT was computed by the arithmetic mean of the last three reversals.

### tRNS Procedure

Using a battery-driven stimulator (NeuroConn, Ilmenau, Germany) tRNS was applied via two rubber electrodes, each in a synthetic sponge that had been soaked in a 0.9% saline solution. The two 5 × 7 cm stimulation electrodes were placed horizontally over T7 and T8 according to the 10–20 system for EEG electrode placement. With a 10 s fade in/out sequence high frequency tRNS (100–640 Hz) was applied. Impedance was kept below 15 kΩ. The stimulation intensity was set to 1.5 mA for all participants. In the sham condition, the current was turned off after 30 s. Debriefing after performing both sessions revealed that participants were not able to correctly indicate in which of the two sessions the tRNS-stimulation was applied.

### Experimental Procedure

**Figure 1:**
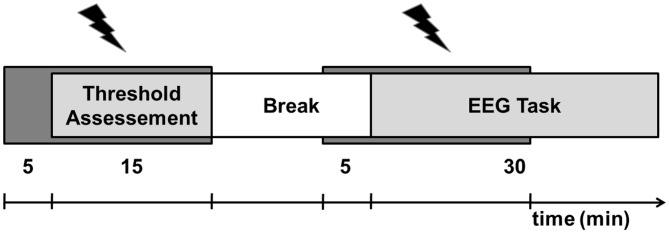
Experimental procedure. The experiment consisted of two sessions (transcranial random noise stimulation (tRNS), sham) recorded on two separated days. Each session started with 5 min of tRNS/sham application (dark gray bar) followed by the alternating assessment of the gap detection threshold (GDT) and the pitch discrimination threshold (PDT) for 15 min. After a short break, tRNS application was again turned on and with a delay of 5 min. the participants performed a gap task and a pitch task twice in alternating sequence while EEG was recorded.

In order to investigate the neurophysiological consequences of the auditory tRN stimulation, we acquired the EEG while participants received either verum or sham stimulation and performed the two domain-specific detection tasks (Figure 1). In the “gap task”, participants performed a two alternative forced choice task with a reference sound without a gap and one of three different gap stimuli: (I) a stimulus clearly above the individual perception threshold with a gap duration of 100 ms representing a high SNR stimulus (SNR_high); (II) a stimulus clearly below the individual perception thresholds with a gap duration of 20 ms, representing a low SNR stimulus (SNR_low); and (III) a stimulus with a gap duration adjusted to the individual GDT, representing a critical SNR stimulus (SNR_crit). Participants were presented with trials sequentially comprising the reference sound and one of the three gap stimuli and had to decide whether the first or second stimulus contained the gap by pressing the corresponding button with their right index finger. A total of 96 trials per condition (SNR_high, SNR_low, SNR_crit) were presented in randomized order with an inter stimulus interval of 1250 ± 125 ms. A similar procedure was used in the “pitch task” with a reference sine wave tone of 1000 Hz and sine wave tone at three different SNRs: (I) with a pitch of 1030 Hz representing a high SNR; (II) a pitch of 1005 Hz representing a low SNR; and (III) a stimulus basing on the individual threshold as assessed in the PDT procedure.

In two different sessions (verum, sham), participants performed the gap task as well as the pitch task twice in alternating sequence resulting in a total of four runs. The order of the sessions and performed tasks within each session was counterbalanced. To avoid fatigue and/or loss of concentration a short break of 2 min was included following the second task. The overall duration of one session was about 32 min. To avoid carry-over effects of the stimulation, there were at least 6 days between the two experimental sessions. During the experiment, participants were seated in a comfortable recliner. Participants were instructed to keep their eyes open during the experiment and to fixate the screen. Task instructions were presented in white font on a black screen. Stimulus material was presented via headphones (Sennheiser HD 65 TV) at 65 dB SPL using the Presentation software, Version 18.1^1^.

Using a single-blinded design, the tRNS application started 5 min prior to the threshold evaluation and lasted for another 15 min. Since the duration of the electrical stimulation (e.g., Miniussi et al., 2013) and the task performed during stimulation (Ruhnau et al., 2016) critically influences its effect the duration of tRNS application was held constant over all subjects. Accordingly, participants performed an individual number of sessions depending on their pace. Subsequently, all participants received a break of 15 min to avoid fatigue and loss of concentration. Five minutes before the end of the break, tRNS was again started and applied for a total duration of 20 min while the EEG-task was performed. Finally, after completing the experiment, participants were asked to answer a short questionnaire consisting of 12 items about their physical state (e.g., headaches, nausea, fatigue, sensation on the scalp, dizziness, phosphenes) during and after the stimulation. Responses were assessed using a four level Likert-scale from 0 (no sensation) to 4 (severe sensation). The statistical analysis of this questionnaire using non-parametric Mann-Whitney-Tests revealed no systematic effect of the stimulation procedure on the physical state of our participants. Thus, tRNS-related differences in alertness, fatigue and/or physical sensations (e.g., itching, heat sensation) could be excluded.

### EEG Acquisition and Data Pre-Processing

**Figure 2:**
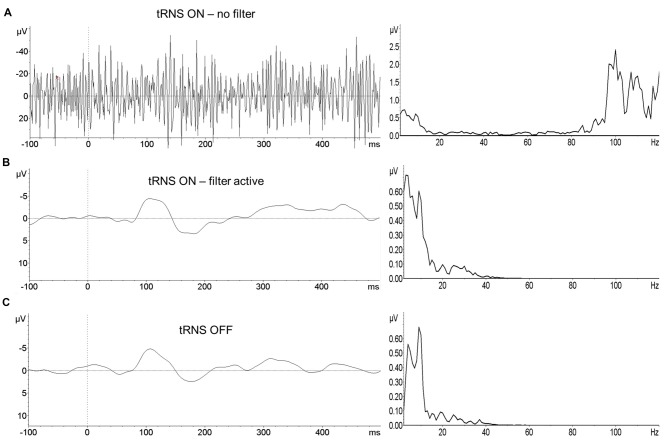
Event related potentials (ERPs) of one subject depicting the mean activity during the gap task. ERPs are recorded at the electrode Cz during tRNS **(A)**, after bandpass filtering the artifact-contaminated signal **(B)** and without tRNS **(C)**. Left panel represents the EEG signal, right panel the corresponding frequency spectrum (FFT).

Simultaneous to the tRNS application, the EEG data was continuously recorded using 3 Ag/AgCl-electrodes at Fz, Cz and Pz according to the international 10–20 system of electrode placement using a BrainAmp DC-amplifier (BrainVision Recorder 1.20; Brainproducts, Munich, Germany). The reference electrode was placed at the tip of the nose, the ground at AFz. Vertical and horizontal eye movements were monitored from electrodes lateral and below the left eye. The impedance was kept below 10 kΩ. The EEG signal was sampled at 1000 Hz, amplified in the range of 327.50 mV at a resolution of 10 μV. Due to the spatial separation of the tRNS electrodes and the EEG electrodes the magnitude of this artifact was limited excluding a clipping of the EEG amplifier. Offline, the data were bandpass filtered between 1–30 Hz (3 dB cutoff frequencies Butterworth filter with zero phase shift) using the BrainVision Analyzer software (Version 2.1.0.327, Brainproducts, Munich, Germany). This procedure allowed removing artifacts of the tRNS stimulation (applied in the range of 100–640 Hz) from the EEG data (Figure 2 illustrates this processing on the basis of exemplary EEG data of one subject). Trials containing eye movements or other artifacts with amplitudes greater than 100 μV were automatically rejected, resulting in a mean number of 75 trials per subject and condition used for the statistical analysis. The processed data were segmented, baseline corrected relative to the 100 ms to 0 ms pre-stimulus time, and averaged for each participant, stimulation condition (tRNS, sham), stimulus SNR (low, high, critical) and condition (gap, pitch), separately. In addition, grand means were computed by averaging across all participants for each stimulation condition and stimulus type, separately. Event-related potentials (ERP)-peak analysis was performed on single-subject averages measured at the vertex electrode (Cz), since this electrode evoked the largest deflections in the grand average. The P50 was defined as the positive deflection following the stimulus-onset in a latency window of 30–100 ms and the N1 as a negative deflection in a latency window between 80 ms and 150 ms after stimulus onset.

### Statistical Analysis

*A priori* sample size calculation by means of G*Power (Faul et al., 2007) for repeated measures ANOVA assuming small to medium effect sizes with a required significance level of 5% revealed that a sample of about 20 subjects is appropriate to avoid false-positive results. However, due to outlier performance in the threshold assessment we excluded two participants from the data analysis. Accordingly the statistical analysis was performed on the data of 18 participants (9 female, Mean age = *M* = 27.12, SD = 3.6).

To evaluate the effects of tRNS on the auditory temporal and spectral resolution for each SNR and condition we assessed the individual tRNS-induced alteration by calculating difference scores between the number of correctly identified stimuli in the tRNS- and in the sham condition. Subsequently, data were entered into repeated measures ANOVAs with the within-subject factor *SNR* (high, low, critical). Effect sizes by means of partial eta squared are reported. Subsequently, planned comparisons by means of *t*-tests (Bonferroni-Holmes corrected) were run.

To investigate the effect of tRNS on event related EEG activity during acoustic processing the P50 and the N1 component of the auditory evoked potentials (ERP) were assessed. Analog to the analysis of the behavioral data, individual difference scores for the amplitude and latency of the ERPs were calculated and entered into repeated measures ANOVAs with the within-subject factor *signal-to-noise* (high, low, crit).

## Results

### Threshold Estimation and Task Accuracy

All participants performed at least one session of the GDT (tRNS: *M* = 3.83, SD = 2.09; sham: *M* = 3.44, SD = 1.5) and two sessions of the PDT (tRNS: *M* = 4.17, SD = 1.95; sham: *M* = 3.72, SD = 1.56). Mean threshold values of the multiple sessions were compared by means of dependent samples *t*-tests, separately for the GDT and the PDT. No statistically significant difference in the participants’ individual threshold was found between tRNS and sham stimulation, neither for the GDT (*T*_(17)_ = 1.379; *p* = 0.186), nor for the PDT (*T*_(17)_ = 1.126; *p* = 0.276).

**Figure 3:**
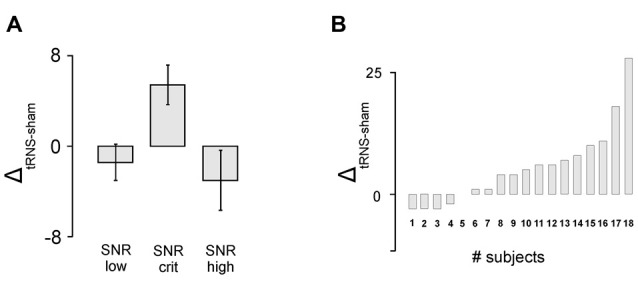
Behavioral results. **(A)** Detection rate in the gap task. tRNS compared to sham lead to increased detection rate for stimuli adjusted to the critical signal-to-noise-ratio (SNR). Error bars represent standard errors (SE). **(B)** Individual data for the critical SNR condition in the gap detection task.

The analysis of detection rate in the gap task revealed a significant main effect of the factor *signal-to-noise* (*F*_(1,16)_ = 3.991, *p* = 0.039, *η*^2^ = 0.333) indicating that tRNS affected the participants’ task accuracy depending on the SNR of the presented stimuli. As depicted in Figure 3 and verified by means of one-sampled *t*-tests, tRNS improved performance for stimuli adjusted to the critical SNR (*T*_(17)_ = 2.911; *p* = 0.010), whereas no such effects were present for stimuli with low (*T*_(17)_ = −0.839, *p* = 0.413) or high SNR (*T*_(17)_ = −1.105, *p* = 0.284). Furthermore, dependent-samples *t*-tests revealed that the tRNS related increase in detection rate for stimuli at a critical SNR (*M* = 5.44, SE = 1.87) was significantly enhanced compared to stimuli with low SNR (*M* = −1.39, SE = 1.65; *T*_(17)_ = −2.467; *p* = 0.025) and high SNR (*M* = −3.0, SE = 2.71; *T*_(17)_ = 2.523; *p* = 0.022). With regards to the pitch task, no significant effects were measured (*F*_(1,16)_ = 0.379, *p* = 0.691, *η*^2^ = 0.045). Detailed information on the mean number and delta values of the correctly identified stimuli can be found in Table 1.

**Table 1:** Behavioral data.

	Low SNR	Critical SNR	High SNR
tRNS (gap)	46.7 (2.57)	51.2 (3.31)	65.8 (10.18)
sham (gap)	47.3 (2.45)	46.2 (2.25)	68.7 (9.73)
Δ tRNS—sham (gap)	−1.3 (1.65)	5.4 (1.87)	−3.0 (2.71)
tRNS (pitch)	54.6 (6.67)	57.4 (4.66)	80.7 (5.29)
sham (pitch)	54.0 (3.44)	57.1 (4.05)	81.9 (5.02)
Δ tRNS—sham (pitch)	0.1 (1.57)	−0.2 (3.18)	−2.0 (2.57)

*Mean number of correctly identified stimuli and delta values (difference score transcranial random noise stimulation (tRNS)—sham) separately reported for the three signal-to-noise-ratio (SNR) levels (SE)*.

### EEG Data

#### P50 Component

##### Peak latency

**Figure 4:**
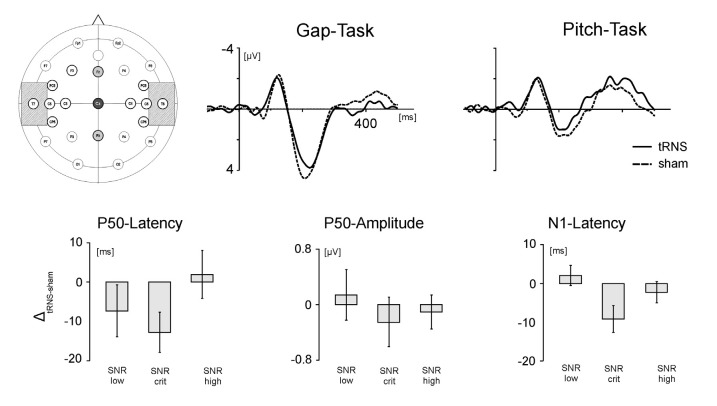
Electrode positioning and EEG results. Upper panel. Left: positioning of the tRNS electrodes over T7 and T8 (crosshatched) and the EEG electrodes over Fz, Cz and Pz. Middle: grand average ERPs during processing of stimuli adjusted to the critical SNR in the gap task. Right: grand average ERPs during processing of stimuli adjusted to the critical SNR in the pitch task. Solid lines represent activation in the tRNS condition, dashed lines activation in the sham condition. Lower panel: tRNS compared to sham modulation of the auditory ERPs for stimuli with low SNR, critical SNR and high SNR. From left to right: latency of the P50 component, amplitude of the P50 component and latency of the N1 component. Error bars represent standard errors (SE).

Figure 4 illustrates the relative effect of tRNS on the P50 latencies of the auditory evoked potentials during the gap-task. Although the ANOVA revealed no significant main effect of the factor signal-to-noise (*F*_(1,16)_ = 1.681, *p* = 0.229, *η*^2^ = 0.168) the data depicted in Figure 4 might indicate that tRNS also modulated the P50 latencies depending on the SNR; analog to the effect observed in the behavioral data (see Figure 3). This observation was underpinned by means of one-sampled *t*-tests revealing that tRNS compared to sham decreased the peak latency when participants were presented with stimuli at the critical SNR (*T*_(17)_ = −2.919; *p* = 0.010). No such effect was present for stimuli with low and high SNR. In addition, the tRNS induced decrease in P50 latency for the critical SNR stimuli (*M* = −15.2, SE = 5.21) tended to be stronger than for stimuli with high SNR (*M* = 0.333, SE = 6.54; *T*_(17)_ = −1.980; *p* = 0.064). Regarding the peak latency in the pitch task, no significant effects were measured (*F*_(1,16)_ = 1.511, *p* = 0.251, *η*^2^ = 0.159) and no difference between the different SNR stimuli reached significance. Detailed information on the mean amplitudes and latencies of the P50 can be found in Table 2.

**Table 2:** Data of the P50 component.

	Latency			Amplitude		
	Low SNR	Critical SNR	High SNR	Low SNR	Critical SNR	High SNR
tRNS (gap)	57.1 (8.64)	53.3 (6.53)	59.5 (7.13)	0.88 (0.60)	0.99 (0.47)	0.41 (0.50)
sham (gap)	64.4 (7.77)	68.5 (8.12)	59.2 (9.03)	0.74 (0.75)	1.24 (0.54)	0.51 (0.64)
Δ tRNS—sham (gap)	−7.3 (6.76)	−15.2 (5.21)	0.3 (6.54)	0.14 (0.37)	−0.25 (0.37)	−0.10 (0.25)
tRNS (pitch)	55.4 (9.53)	57.8 (10.77)	59.7 (8.26)	1.16 (0.44)	0.52 (0.49)	0.80 (0.51)
sham (pitch)	67.6 (6.47)	66.4 (7.6)	57.9 (7.87)	1.16 (0.59)	1.12 (0.47)	1.083 (0.64)
Δ tRNS—sham (pitch)	−12.1 (4.04)	−8.6 (5.26)	1.8 (3.87)	−0.002 (0.27)	−0.61 (0.34)	−0.28 (0.30)

*Mean latencies (in ms) and amplitudes (in μV) for the correctly identified stimuli and delta values (difference score tRNS—sham) of the P50 component separately reported for the three SNR levels (SE)*.

##### Peak amplitude

No statistically significant differences were found for the peak amplitude of the P50 component. This holds true both for stimuli representing the temporal as well as the spectral acoustic domain. However, on a descriptive level, Figure 4 shows that the effect of tRNS was modulated by the SNR of the processed stimuli. The P50 amplitudes during the processing of gap stimuli at the critical SNR were diminished by tRNS while there was no stimulation effect for the processing of stimuli with low and higher SNR. This suggests that the peak amplitudes in the gap task follow the same inverted U-shaped form as the behavioral parameters and the P50 latency. No such SNR-specific pattern for the peak amplitudes was found in the pitch task.

#### N1 Component

##### Peak latency

Analysis of N1 latencies during the gap task revealed a significant main effect of the factor *SNR* (*F*_(1,16)_ = 4.413; *p* = 0.030, *η*^2^ = 0.356). Thus, tRNS modulated the latency of the N1 component depending on the SNR of the presented stimuli (Figure 4). One-sampled *t*-tests revealed that whereas tRNS decreased the peak latency when participants were presented with stimuli at the critical SNR (*T*_(19)_ = −2.509; *p* = 0.023), no tRNS effect was present for stimuli with low and high SNR. Moreover, the tRNS related decrease in the N1 latency for critical SNR stimuli (M = −8.72, SE = 3.47) was significantly stronger than for stimuli with low SNR (*M* = 2.17, SE = 2.60; *T*_(17)_ = 2.189; *p* = 0.043). No significant stimulation effects were measured in the pitch task (*F*_(1,16)_ = 0.438; *p* = 0.653, *η*^2^ = 0.052). Detailed information on the mean amplitudes and latencies of the N1 can be found in Table 3.

**Table 3:** Data of the N1 component.

	Latency			Amplitude		
	Low SNR	Critical SNR	High SNR	Low SNR	Critical SNR	High SNR
tRNS (gap)	121.7 (7.23)	113.1 (8.72)	114.9 (9.14)	−3.20 (0.87)	−2.35 (0.69)	−2.78 (1.08)
sham (gap)	116.1 (7.37)	123.0 (7.16)	117.8 (8.27)	−3.88 (1.14)	−3.53 (1.14)	−3.84 (1.33)
Δ tRNS—sham (gap)	2.2 (2.60)	−8.7 (3.47)	−2.1 (2.49)	0.67 (0.50)	1.18 (0.64)	1.06 (0.35)
tRNS (pitch)	118.0 (9.43)	116.7 (9.71)	122.2 (10.13)	−2.46 (0.88)	−2.22 (0.85)	−2.30 (0.94)
sham (pitch)	116.8 (8.59)	119.1 (9.59)	120.7 (6.47)	−2.58 (0.87)	−2.67 (0.92)	−2.44 (0.93)
Δ tRNS—sham (pitch)	−2.4 (4.17)	−1.0 (4.52)	3.8 (4.21)	0.12 (0.48)	0.45 (0.442)	0.14 (0.56)

*Mean latencies (in ms) and amplitudes (in μV) for the correctly identified stimuli and delta values (difference score tRNS—sham) of the N1 component separately reported for the three SNR levels (SE)*.

##### Peak amplitude

No statistically significant effects were found for the peak amplitudes of the N1 component, neither for the gap, nor for the pitch task.

Taken together, for the temporal domain (gap detection task) we found a facilitating effect of tRNS on processing stimuli with a critical SNR while the application of tRNS had no beneficial effect on the processing of stimuli at a low or high SNR. This behavioral finding was paralleled by an acceleration of the P50 and the N1-component. Both, the behavioral and electrophysiological effects were limited to processing stimuli at a critical SNR, while no modulation of lower or higher SNR stimuli occurred. tRNS had no effect on the processing of spectral information.

## Discussion

The present study investigated the impact of tRNS over the bilateral auditory cortex on participants’ temporal and spectral auditory resolution ability. Behavioral acuity in domain-specific detection tasks and related electrophysiological correlates were measured while the participants were presented with stimuli representing a critical SNR as well as with stimuli of low and high SNR. We found a beneficial effect of tRNS in detecting gap stimuli at the critical SNR only, while no effect was present for stimuli with low and high SNR. This finding was paralleled by reduced peak latencies in the N1 component and, on a descriptive level in the P50 component, indicating that the behavioral results rely on facilitated sensory bottom-up processing in the auditory cortex.

The general efficacy of tRNS to modulate the auditory cortex resonance frequency has been demonstrated previously by Van Doren et al. (2014) showing increased gamma oscillations in Heschl’s gyrus after bilateral tRNS. Our present findings extend the current knowledge on auditory tRNS by demonstrating the behavioral manifestation of the externally amplified auditory resonance frequency. In concrete, our data show an improvement in processing time critical acoustic information, presumably caused by an increase in the individual resonance frequency of the auditory cortex. Moreover, we found tRNS-induced modulations of event-related electrophysiological measures. In sum, tRNS allows for a transient enhancement of auditory perception.

As hypothesized, in the present study we demonstrate improved detection rate for stimuli at threshold critical SNR only, while no beneficial effect on high and low SNR stimuli was evident. This finding of a specific tRNS effect for stimuli with a critical SNR further suggests that tRNS administered to the auditory cortex modulates the reactivity of the stimulated region via SR. SR is a ubiquitous phenomenon in non-linear systems characterized by improved detection rate of near-threshold information when an optimal level of noise is induced in the system (Longtin, 1993; McDonnell and Abbott, 2009). The relationship between the detection rate and the neural noise, however, follows an inverted U-shaped function: compared to a zero-noise condition perception is impaired at an SNR both at the lower and the upper end of the scale but enhanced when an optimal level of noise is present in the system (Moss et al., 2004). In the auditory system, psychophysical studies demonstrate this non-linear relationship in (animal) models (Wiesenfeld and Moss, 1995; Henry, 1999), in patients with cochlea or brainstem implants (Morse and Evans, 1996) as well as in perception of non-speech and speech stimuli in healthy subjects (Zeng et al., 2000; Ward et al., 2010).

According to signal detection theory (Nevin, 1969), the detection rate of a given stimulus depends on the specific ratio between signal power and noise power (i.e., the signal to noise ratio). Accordingly, in our study, we modified the SNR by means of the task difficulty. Participants were presented with stimuli clearly above the perception threshold (high SNR stimuli), stimuli clearly below the perception threshold (low SNR stimuli) and stimuli adjusted to the individual detection threshold (critical SNR stimuli). We found that only stimuli adjusted to the individual detection threshold are susceptible to tRNS-induced SR. Analog SR-like tRNS-effects have been already demonstrated for improvements in arithmetic reasoning (Popescu et al., 2016). In this study tRNS mitigated the effect of task difficulty on the participant’s response time. In particular, tRNS was effective to modulate stimuli with a critical SNR (difficult problems) only, while no effect was found for stimuli with high SNR (easy problems). Finally, the required noise can also be characterized by means of acoustic stimulation and using electrical pulses (Iliopoulos et al., 2014). In the visual system, van der Groen and Wenderoth (2016) recently provided evidence that adding visual noise or applying electrical noise via tRNS over the visual cortex both modulates perception according to the SR-typical inverted U-shaped relationship between noise and detection rate.

A second line of reasoning not mutually exclusive with the SR-argument is increased synchronization within and between feature-relevant neural networks. Synchronization, as reflected by synchronous oscillations among a large number of pyramidal neurons, results in event related potential in the scalp EEG. Typically, with increasing number of neurons involved, the amplitude of the ERP increases. Ward et al. (2010) measured enhanced brain responses to 40 Hz pure tones when the optimal amount of noise i.e., randomly varying broadband acoustic noise was applied. In the same vein, Vanneste et al. (2013) proposed that tRNS normalizes the usually present hyper-synchronization in auditory cortex regions of patients suffering from tinnitus. This assumption has been confirmed by two recent studies demonstrating that the application of tRNS reduces the subjectively perceived tinnitus loudness and tinnitus related distress (Vanneste et al., 2013; Claes et al., 2014; Joos et al., 2015). However, in our study, we measured reduced peak latencies rather than modulations in the peak amplitude. Response latencies have been shown to reflect neural conduction time (Lister et al., 2011), implicating that tRNS facilitates the firing of neuron population typically involved in the relevant processes rather than it increases the number of involved neurons. One might also speculate that tRNS positively influences the refractory time exhibited by neurons in the auditory cortex.

Since oscillations in the gamma range are the dominant rhythmic activation pattern in the auditory cortex (Giraud et al., 2007; Gross et al., 2013; Baltus and Herrmann, 2015) and this oscillation pattern is strongly associated with the processing of rapidly changing acoustic features (Poeppel, 2003; Giraud and Poeppel, 2012), it seems plausible that auditory tRNS amplifies the inherent resonance frequency resulting in increased neural SNR and improved perception of time-critical acoustic information. This notion is underpinned by the null result on the processing of spectral acoustic features, a function typically associated with neural oscillations in the theta band (4–8 Hz; Luo and Poeppel, 2012). Although the exact neurophysiological implementations of both temporal and spectral acoustic processing remain a matter of debate (Patterson et al., 2002; Hall and Plack, 2009; Plack et al., 2014), there is reasonable evidence on neuron populations in the auditory cortices specialized in processing temporal or spectral auditory features, respectively (Zatorre and Belin, 2001; Poeppel, 2003; Giraud and Poeppel, 2012). The proposed feature-specific focus is manifested by two different oscillation patterns (gamma and theta) aligning the acoustic stream and parsing it into information units of appropriate granularity. This functional specialization has been suggested to rely on micro- and macro structural properties of auditory cortex regions (Hutsler and Galuske, 2003; Jung-Beeman, 2005). Even though tRNS has been proposed to stimulate neurons irrespective of their spatial orientation (Terney et al., 2008) our domain-specific findings might also rely, at least in part, on the different susceptibility of the functionally specialized neuron populations (Radman et al., 2009).

Although the results of our study are limited to normally hearing participants, our findings have implications for the clinical application. Features in the temporal domain mainly determine linguistically relevant information in the speech signal. Phonemes, the smallest meaningful units in language, for instance are characterized by VOT and formant transition. Accordingly, impaired temporal processing is considered as the underlying deficit in developmental dyslexia but also in age-related hearing loss. The auditory system, a strictly non-linear entity (Eguíluz et al., 2000), uses noise to increase the inherent SNR (Henry, 1999; Zeng et al., 2000; Hedrick et al., 2016). Adding noise enhances pure tone detection threshold in patients with cochlea and brainstem implants (Zeng et al., 2000). In the animal model, improved vowel detection was achieved when applying electrical noise (Morse and Evans, 1996). Jaramillo and Wiesenfeld (1998) suggested that even the inner hair cell might benefit from noise-induced SR. Since pathologies of inner hair cells are the most common cause of age-related hearing loss it seems appropriate to investigate whether tRNS improves auditory perception in older adults. Moreover, the frequency unspecific mechanism of tRNS allows the application without the prior knowledge of the endogenous target frequency and, thus, even further enhances the applicability of this stimulation technique in the clinical setting.

### Limitations

The main aim of the present study was to assess the effect of tRNS on auditory resolution ability by inducing SR. As hypothesized, we found a beneficial effect of tRNS on the detection rate for near threshold stimuli in the temporal domain. However, no such effect was present in the threshold assessment, although tRNS was applied during both parts of the experiment. Improved detection rate as the consequence of the SR mechanism can only take place for the near threshold signal, thus, for a signal with a critical SNR. No such effect can be expected for stimuli with lower or higher SNR. We therefore assessed the individual perceptual threshold as valid as possible, thus, while tRNS was administered. With this procedure we controlled that in the following EEG task each participant was presented with: (I) a stimulus clearly above the individual perception threshold (SNR_high); (II) a stimulus clearly below the individual perception thresholds (SNR_low); and (III) a stimulus with a gap duration adjusted to the individual GDT (SNR_crit). Since tRNS was applied both during the threshold assessment as well as during the EEG task our study design does not allow drawing conclusions on the time course of tRNS effects on auditory resolution. In the same vein, the Null result in the threshold assessment might represent either the limited susceptibility to tRNS or the (insufficient) duration of the electrical stimulation.

Finally, in the present study we used an electrode placement which: (I) has been shown to affect auditory core regions most strongly using modeling (Neuling et al., 2012); and (II) was applied successfully in previous studies on auditory processing (Van Doren et al., 2014; Joos et al., 2015; Rufener et al., 2016a, b). By subsequently comparing the effects of tRNS and sham stimulation, our experimental procedure allowed for a systematic evaluation of the effect of auditory tRNS on the processing of speech-relevant acoustic features. However, our study did not investigate the influence of different tRNS electrode montages on acoustic processing and, thus, spatial specificity of the tRNS application was not additionally manipulated in the current setting. Future studies are needed to shed more light on how factors such as e.g., stimulation time and electrode location modulate tRNS effects on auditory perception, but also on cognitive functions.

In sum, the present study demonstrates for the first time the efficacy of tRNS to improve the processing of temporal acoustic features, presumably via enhancement of endogenous auditory gamma oscillations. The specific effect on stimuli at the critical SNR provides further evidence for SR as the underlying mechanism of our findings.

## Author Contributions

TZ and KSR conceived and designed the experiments; analyzed the data. KSR performed the experiments. TZ, KSR and PR wrote the article. H-JH and PR contributed reagents/materials/analysis tools.

## Conflict of Interest Statement

The authors declare that the research was conducted in the absence of any commercial or financial relationships that could be construed as a potential conflict of interest.

## Footnotes

^1^www.neurobs.com
